# Supplementary material for: Patterns of Intron Gain and Loss in Fungi
Source: PLoS Biol. 2004 Nov 30;2(12):e422. doi: 10.1371/journal.pbio.0020422 (PMC532390; doi:10.1371/journal.pbio.0020422)
Supplement: Table S1 — Also available at http://genes.mit.edu/NielsenEtAl/. (4.3 MB ZIP). [file pbio.0020422.st001.zip › NielsenEtAl/html/4.html]

AN2441.1.NCU08040.1.MG01832.1.FG04268.1


```
 CLUSTAL W (1.82) Multiple Sequence Alignments - Introns Inserted


Sequence 1: NCU08040.1	547 aa
Sequence 2: MG01832.1	534 aa
Sequence 3: FG04268.1	533 aa
Sequence 4: AN2441.1	554 aa
Alignment Length: 569 aa
Number Identitical Residues: 200 aa
Alignment Score (without introns) 10588


MG01832.1 	MTEVIMTQTPPVLNGPSEKEKKYDRQLRLWAASGQAALESANILLVSSG----------A
NCU08040.1	MTEIVMDQTPPILQGPSDKERKYDRQLRLWAASGQAALESANILLVNSG----------A
FG04268.1 	-MADVMSQTPPVLHGPSEKERKYDRQLRLWAASGQAALESANILLVNSG----------A
AN2441.1  	----MTSEFPHALTGPSSKERKYDRQLRLWAASGQQALEESRVLLVNSDGPWGNRSTGVS
          	    :  : *  * ***.**:************** ***.:.:***.*... .. ::. :

MG01832.1 	GTVGVETLKNLVLPG1IGQFTIYDPATVCESDLGVNFFLDEDSLGKSRAQCCTEMLLELN
NCU08040.1	GTVGVETLKNLILPG1IGRFVIHDNALVDEADLGVNFFLDDSCYGKPRAQCLASLLGELN
FG04268.1 	GTVGVETLKNLVLPG~IGQFTIADKSVVGYEDLGVNFFIDDSWLGKSRAEACTNFLLELN
AN2441.1  	GVVGVETLKNLVLPG~VGGFTIVDPAVVTEPDLGVNFFLENESLGRSRAEETCRLLKELN
          	*.*********:*** :* *.* * : *   *******:::.  *:.**:    :* ***

MG01832.1 	PEVHGEWHPN~SE0SGALTLAQLLEKSP----TFTMIIYSHPITEADKDLLWTYGSKHKT
NCU08040.1	PEVDGDWSPK~TK0ND--SLGSLLKQSP----LFTAIMYTYPINHVELETLEQYSKEHKT
FG04268.1 	PEVQGEWYPK~NQ0GESFDLEAFLSDSP----IFTMILYALPLPQDQVQLIQNYAHKHSI
AN2441.1  	PDVEGSFRTK0--~----PIAEILQEEPGILAQHKLILISGPIEHSSLRALCDGAKKLAI
          	*:*.*.: .:         :  :*...*.  : .. *: : *: . .   :   . :   

MG01832.1 	PLISMHSAGFYSYFQVKLPGAFPIVDTHPDETATTDLRLLTPWVELQQFAKELTYNIDSL
NCU08040.1	PLIAIHSTGFYSYFTIRLPGTFPIVDTHPDETATTDLRLLSPWPELVEFANTMTKDIDSL
FG04268.1 	PTISVHSVGFYSYFKSTLPGTFPIVDTHPDETATTDLRLLAPWPELVEFSRGMTENIDTL
AN2441.1  	PVLYTRSVGFYSTFSLQLPAVFPIVETHPDPESTQDLRLLNPWPELTAAG-ASIRNLDSF
          	* :  :*.**** *   **..****:****  :* ***** ** **   .     ::*::

MG01832.1 	DDHEHGHLPYVAILLHYLDQWRDAHEGRYPTTYAEKKEFRTLVSQGARIGNATGPEENFE
NCU08040.1	DNFEHGHLPYVAILLHYLDKWKATHNGTYPSTYKEKTEFRQLVRDAARTDNPEGGEENFD
FG04268.1 	DNHEHGHLPLVVILLHYLEQWQQTHDGAYPTSYADKTSFRKTVSEAMRTDNPEGGEENFE
AN2441.1  	DDHQHGHVPYVLLLLHYLEKWKETHNGSVPSNYKEKTAFRDFVRSCARTNNSQGGEENYD
          	*:.:***:* * :*****::*: :*:*  *:.* :*. **  * .  * .*. * ***::

MG01832.1 	EAVAAVLKTISPPSLPDGLKEVFRYLDSHK0TEERTG-FWLIAAAIREFWEKNKCLPVPG
NCU08040.1	EAAAAVLKTVAPFSLPSGLKEVFEYEHKDP0IQERST-FWIIADAVKAFYTKHGSLPLPG
FG04268.1 	EAVAAVMKHVVTPSLPSSLQQVFDFKHQDP0EETKSS-FWIIAEAVKRFYNEHSRLPVPG
AN2441.1  	EAVAAVLKSLNPFSLRSSTREIFEMEECRQ~LSAASADFWVIASAVREFYETHQLLPLPG
          	**.***:* : . ** .. :::*   .     .  : .**:** *:: *:  :  **:**

MG01832.1 	KVPDMKAQSNVYVRLQNIYKSKARKDVAEVLDIVR---TYPG-GKEVDPSGVELFCKNAA
NCU08040.1	NVPDMKAQSKVYVQLQNIYKAKARKDAAEVLQTAR---AIAGTGREVDPAEVDLFCKNAA
FG04268.1 	GLPDMKAQSSVYIKLQNIYKERARQDVSQVLETVR---GIPG-GEDVDPEQIELFCKNAR
AN2441.1  	SLPDMKAQSADYVSLQNIYKTKAREDIAEVTDIIRRLESQLGRAARVDDKDIEIFCKNAS
          	 :*******  *: ****** :**:* ::* :  *  .   * .  **   :::***** 

MG01832.1 	FVKLINAAEDGTNG-----DRLAKVVE1DELANDAIAEAS--MLPLSLVPIYFALSVTAH
NCU08040.1	FVKLINVEGGGDTAPLGSKERLQQVLR1QEMANDQVAEMT--LQPMSLFPIYLALRGLAH
FG04268.1 	FIKLINSPEERAVK-------LDQVVE1QQLANDEIAAMAGPEMPLSLIPLYLALLASSN
AN2441.1  	HIQVIRGRDIPQVD---GGEDTKKRLR~LELNNDESLIPVY-LAFEVLDTVVTGIQEGKY
          	.:::*.           . .   : :.  :: **             * .:  .:     

MG01832.1 	DPTATR------EQIMSAIKDCVPS--VGDH-ERLLEASEEVARAGGAELHNISALTGGM
NCU08040.1	CGNASSDKTQIVEAVTSLLSDNLSSEEYGDLNEKLGNVAEDLARAEYGELHNISALTGGM
FG04268.1 	STTATV------DEIMAFISINAPQ--AADN-ERYKKTAQELERAAGGELHNISALTGGM
AN2441.1  	HQDALDDDGIWSSEIGRILAVIAADAAVDEARNRVLDAAQELRRTKGGELHNISSLTGGL
          	   *  ..    . :   :    ..    :  ::  ..:::: *:  .******:****:

MG01832.1 	VAQEMIKIITKQYIPVDNTCVFDGIGSRCQTLRL
NCU08040.1	VAQEMIKIITKQYIPIDNTCVFDGIRSRTQVFHV
FG04268.1 	VAQEMIKIITKQYVPIDNTCIFDGIDSRCQVLRL
AN2441.1  	VAQEALKVLTRQYVPLDNTCIFDGVRSSSAMYKL
          	**** :*::*:**:*:****:***: *     ::
```
